# Retraction: a novel combination of Chinese medicines to treat advanced cancers and lymphomas tested in rats

**DOI:** 10.1186/1749-8546-5-10

**Published:** 2010-03-16

**Authors:** Dawn N Waterhouse

**Affiliations:** 1 Department of Advanced Therapeutics, BC Cancer Research Centre, 675 West 10thAvenue, Vancouver, BC V5Z 1L3, Canada

## Abstract

The author has withdrawn this article [1] from the public domain because they did not have permission to use the data that was presented within. In the light of this situation, BioMed Central regrets that this article is no longer available. The author apologises to all parties for the inconvenience.
